# On the coincidence of weather extremes and geopolitical conflicts: Risk analysis in regional food markets

**DOI:** 10.1371/journal.pone.0323379

**Published:** 2025-05-28

**Authors:** Nkongho Ayuketang Arreyndip, Jeremy S. Pal

**Affiliations:** 1 Department of Environmental Sciences, Informatics, and Statistics, Ca’ Foscari University of Venice, Venice, Italy; 2 CMCC Foundation - Euro-Mediterranean Center on Climate Change, Economic Analysis of Climate Impacts and Policy Division, Venice (VE), Italy; 3 RAAS-CMCC Risk Assessment and Adaptation Strategies, Institute of Climate Resilience, Lecce, Italy; National Taiwan University, TAIWAN

## Abstract

Given the recent increase in geopolitical tensions between major agricultural producers and weather extremes, there is a likelihood that geopolitical conflict will occur simultaneously with weather extremes, leading to devastating production losses between the conflicting parties. These losses can affect the entire food supply chain, leading to food shortages and price increases in regional markets. This paper models the impact of these concurrent events on the global food market, using the Russian-Ukrainian war and the extreme heatwaves of summer 2022 as a case study. The model considers four war scenarios: the start of the invasion, the peak of the war, Ukraine’s resistance, sanctions against Russia, and refugee crises in Europe. Using data from the US Department of Agriculture (USDA), Statista, WITS, and Acclimate production value losses, the results show that the agricultural sectors of southern European countries such as France, Italy, and Spain were the most affected by the extreme events, although the direct impact of refugees was smaller compared to their northern counterparts. Strict sanctions against Russia coupled with Ukraine’s resistance will benefit EU food markets, but at the same time the agricultural sectors of smaller countries and weaker economies, particularly those of Russia’s allies, will be highly vulnerable. This study suggests that when developing and adopting conflict resolution strategies, their impact on weak economies should not be overlooked. An example of this policy recommendation is the continuous renewal of the Black Sea Grain Initiative to stabilize global food prices.

## 1 Introduction

Russia’s full-scale invasion of Ukraine (February 24, 2022) triggered an unprecedented global economic crisis and further hampered global efforts to recover from the COVID-19 pandemic. Rising energy and food prices, high inflation rates, economic stagnation, poverty, refugee crises, etc. characterized the consequences of this invasion [1–6]. European economic growth shrank considerably and the euro lost value against the dollar. The European Union (EU), the United States (US), the United Kingdom, Japan, Australia, Canada, and other allies imposed a rapid succession of tough sanctions against Russia following the invasion of Ukraine, such as the exclusion of Russia from SWIFT with the aim of removing Russia completely from the international financial system, the freezing of Russian and Russian assets, a ban on the export of raw materials such as gas, crude oil and grain, a ban on the import of goods to Russia such as war equipment, technological devices, food, energy, etc [5–10]. These sanctions were also extended to Russia’s allies such as Belarus and Iran [6]. On the other hand, Ukraine has received massive support from the EU, the US, the UK, and other allies in the form of military equipment, solidarity funds, medical care and food, refugee resettlement programs, pledges for Ukraine’s future reconstruction, and the possibility of Ukraine’s EU candidacy is imminent [11]. In total, the EU has allocated over 82 billion euros for solidarity with Ukraine as of September 29, 2023 [11].

The economic impact of the war is being felt in all parts of the world [12] and in all sectors, with the eurozone being the hardest hit due to its heavy dependence on Russian energy imports, which accounted for almost 50% of its energy consumption in 2022 [3, 13–16]. As both Russia and Ukraine are major food exporters to the Eurozone, this also triggered massive shocks in food markets, leading to empty shelves in supermarkets, panic buying, and rationing, as well as unprecedented price hikes, which rose to a staggering 14.1% year-on-year in January 2023 [3]. Inflation reportedly rose from 2.6% in 2021 to 8.4% in 2022 [3, 17, 18], while unemployment also rose to 6.6% in December 2022 [3]. According to the United Nations High Commissioner for Refugees [19], almost 7 million Ukrainian refugees were counted worldwide by November 21, 2023, including over 5 million in Europe. The influx of refugees into the rest of Europe could lead to housing crises, food shortages, and an increase in the cost of living [20]. Nevertheless, these migrants represent a great opportunity for future economic growth due to the projected increase in the EU labor force, consumption, and trade [20].

In July 2022, the Black Sea Grain Agreement was signed between Russia, Ukraine, Turkey, and the United Nations. This initiative was to facilitate the export of grains and fertilizers to the global market as a means to address the global grain shortages and soaring food prices due to the war [21]. The main objective of this initiative was to help stabilize global food prices and prevent famine in vulnerable countries. The initial agreement was to last for 120 days and was extended three times, for 120 days on November 19, 2022, for 60 days on March 18, 2023, and lastly on May 17, 2023, for another 60 days before not reaching any further agreement to renew the deal. By investigating the economic impacts of the black see grain initiative, [22] found approximately $116.05 billion in losses in the global wheat and corn markets. They also found that the Black Sea Grain Initiative reduced prices of Wheat by 7.9%, offsetting approximately $21.48 billion of these costs. The short-term market impacts assessment of four (4) grains under the Black Sea Grain Initiative was carried out by [21] with his findings showing that the events of a positive abnormal return for the agricultural grain markets with the outbreak of the war and the non-renewal of the Black Sea Grain Agreement, led to an increase in the prices of grains.

In Asia, a report by the Asian Development Bank (ADB) shows strong GDP growth in the Caucasus and Central Asia shortly after the Russian invasion of Ukraine [23]. In another ADB report [24], this growth forecast was lowered due to slower expansion in the People’s Republic of China (PRC). According to the African Development Bank (AfDB), significant grain shortages have been reported in Africa, as the continent is a net importer of agricultural commodities [25]. These grain shortages and high inflation rates in local currencies further exacerbate regional food insecurity and poverty. However, North Africa’s and the Middle East’s wheat markets were found to be the least impacted according to the study carried out by [22]. The continent has also been unsettled by the military coup in Niger, as the country has fallen out with France and the West and found new allies in Russia [26].

With the increase in the world population, urbanization, and industrialization, anthropogenic CO2 emissions are also increasing. These anthropogenic activities are associated with an increase in global average temperature, leading to an intensification and recurrence of extreme weather events [27–41]. The agricultural sector, one of the economic sectors most affected by these extreme weather events, as plants rely on water, moisture, sunlight, and temperature for germination, growth, and productivity, has been the subject of much interesting research [42–59]-65] because of the importance of food to the existence of humans, animals, and plants. Furthermore, before this invasion, there were the record-breaking summer heatwaves of 2018-2021, which researchers report have caused numerous crop failures across the mid-latitude region [31–33]. Given the recently reported record-breaking heatwaves in the summer of 2022 in conjunction with the Russia-Ukraine crisis, the short- and long-term cascading economic impacts will be even greater than expected. The economic impacts of single and simultaneous extreme weather events have been extensively studied in the literature [32, 44, 66–68], especially in the global food web [44, 60, 65, 69]. What has never been studied is the response of the global food market to cascading weather and geopolitical extremes, especially when conflict regions are important breadbaskets. The insights gained here could be useful for policymakers working on the effective implementation of the Sendai Framework for Disaster Risk Reduction (2015-2030), the Paris Agreement, and the 2030 Agenda for Sustainable Development.

The continuous Russia-Ukraine war, the recent Israel-Hamas war, and extreme weather events have caused severe damage to the global economy. Regional agricultural sectors are not yet prepared in terms of policies, adaptations, and mitigation strategies to combat these events due to the lack of thorough assessment reports. Using the Russia-Ukraine war and the 2022 summer weather extremes as a case study, the objective of this paper is to assess the risks posed by the unprecedented weather-geopolitical extreme events on regional and global agricultural sectors, and to raise awareness of the devastating economic crises posed by these events, contributing to early policy-making and sectoral intervention to mitigate or prevent future humanitarian crises. The rest of the paper is arranged as follows, in Sect 2 we present the data we have used for the scenario quantification and distribution. We equally present the economic network model and the EORA input-output data used for the shock impacts analysis. A description of our method of shock distribution to mimic the shocks exerted on regional agricultural sectors due to the Russia-Ukraine war and the Summer 2022 extremes. Sects 3 and 4 present the results and discussions of our numerical experiment and we conclude in Sect 5.

## 2 Data and methods

### 2.1 Data

The agricultural production data from the United States Department of Agriculture (USDA) found at https://www.nass.usda.gov/Data_and_Statistics/ (accessed on 20 Nov 2023) for 2022 was used. Analyzing this data during the Russia-Ukraine war period gives us an overview of the impact of this war in key breadbasket regions. Statista’s Ukraine refugee data found at https://www.statista.com/statistics/1312584/ukrainian-refugees-by-country/ has been analyzed to quantify the impact of the Russia-Ukraine war due to Ukraine refugee crises over Europe and globally. Additionally, data from the World Integrated Trade Solutions (WITS) of the World Bank(WB) has been used to analyze the real-time impact of the war on Ukraine’s and Russia’s Cereal export trade flows. The year 2022 was compared to the year 2020 long before the full-blown war. The WITS data can be found at https://wits.worldbank.org/.

The ERA5 surface temperature data from 1960 to the present taken from the Climate Data Store (CDS) of the Copernicus Climate Change Service (C3S) https://cds.climate.copernicus.eu/cdsapp#!/dataset/reanalysis-era5-single-levels?tab=overview, has been employed to examine the 2022 summer anomalies compared to 1991-2022 climatology over Europe and Ukraine and also contribute in the designing of the scenarios. Additionally, we have also made use of the SPEI3 data (https://spei.csic.es/map/maps.html#months=1#month=2#year=2024) from the global drought monitoring system from June to August 2022 to analyze drought impacts.

### 2.2 Economic network acclimate

The economic network we have employed in this study is called Acclimate. Acclimate is an agent-based, demand-driven economic network model that simulates the propagation of shocks in the global supply network. This model can be used to monitor shocks caused by natural and/or man-made disasters. It consists of highly interconnected regional sectors, where the regions represent every country in the world and the sectors are the different industries that make up a country’s economy, e.g. the agricultural sector, food, hotels and restaurants, wholesale trade, oil and gas, timber, transportation, finance, mining, and quarrying, etc [44, 66, 70]. In this work, Acclimate has been used alongside the EORA 2013 input-output dataset. The EORA dataset is a multi-region input-output table at the global level to estimate value added in trade. This dataset describes the annual monetary flows between 26 major sectors and final demand in 188 countries. It covers 15,909 sectors in 188 countries. In this work, we apply the scenarios model to the agricultural sectors of Ukraine, Russia, and the EU which exist as nodes in the network. We then compute the agricultural production value losses during the period of hostilities and compare them to the baseline (no extreme events).

The Acclimate model, developed by a team of scientists at the Potsdam Institute for Climate Impact Research (PIK), in Potsdam, Germany, has been experimented with and found to be very robust in simulating economic shock propagation in the global supply chain network [44, 66–68, 70, 71]. More about the EORA network can be read here https://worldmrio.com/documentation/ while the Acclimate model can be found here https://github.com/acclimate/acclimate.

### 2.3 Shocks quantification and distribution

**Fig 1:**
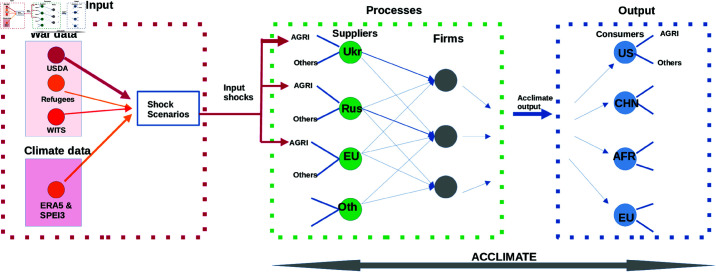
A modeling framework to simulate the propagation of shocks originating from weather-geopolitical coupled extreme events down the food supply chain. Agricultural output data from the USDA, WITS export trade data, and Statista refugees data have been used to quantify the impact of the war while ERA5 weather data has been used alongside the SPEI3 drought data to quantify the contribution of extreme weather events to agricultural production losses. The damage function used to shock the regional agricultural sectors in the Acclimate model has been derived from these input data.

**Fig 2:**
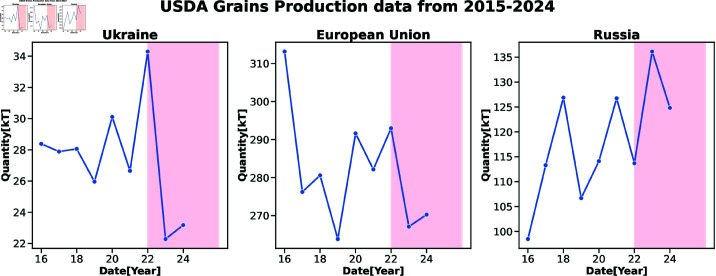
The USDA agricultural production data from 2015 to the present shows the impact of the Russian-Ukraine war on Ukraine, Russia, and the EU. The pink region highlights the period of the war. From 2022 to 2023, the figure shows a significant drop in production output for Ukraine and the EU while Russia’s production quantity shows a significant increase. The values are in kilo tonnes.

**Fig 3:**
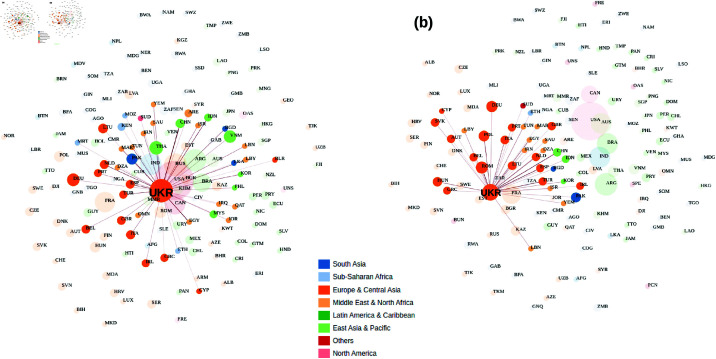
A screenshot of the structure of the WITS Cereal export trade data for Ukraine. WITS Cereal export data of Ukraine for 2020 (a) compared to 2022 (b) shows a drastic drop in the number of trading partners. The size of the bubble indicates the global share of production quantity, the line thickness indicates the quantity of commodity flow, and the color bar shows the continental locations of the export partners. In this figure, we see that due to the war, Ukraine can only export its product to Europe while its production quantity has significantly reduced in 2022 compared to 2020.

**Fig 4:**
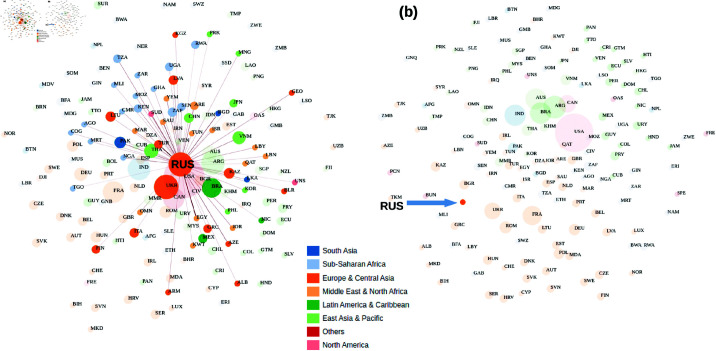
A screenshot of the structure of the WITS Cereal export trade data for Russia. WITS Cereal export data of Russia for 2020 (a) compared to 2022 (b) shows a drastic drop in the number of trading partners and production quantity. The size of the bubble indicates the global share of production quantity, the line thickness indicates the quantity of commodity flow, and the color bar shows the continental locations of the export partners. In this figure, we see that due to the war, sanctions on Russia completely cut off the global Cereal network in 2022 compared to 2020.

To model the impact of weather-geopolitical extreme events on the regional and global agricultural sector, we develop a modeling framework to simulate the propagation of shocks emanating from these events in the food supply chain (Fig 1). To quantify and design the damage function, we analyze USDA agricultural production data as a preliminary assessment of the impact of the Russian-Ukrainian war on agricultural production losses at the country level (Fig 2). The data runs from 2015 to date. This figure shows the impact of the Russian-Ukrainian war on Ukraine, Russia, and the EU. The pink region highlights the period of the war. Comparing the 2022 and 2023 production output data, results show a significant drop in agricultural production output for Ukraine and the EU but an increase in production output is seen in Russia. We equally analyzed the WITS Cereal export data of Ukraine and Russia for 2022 compared to 2020 when there was no war (Figs 3 and 4 respectively). Both the USDA production data and WITS Cereal trade data show a significant drop in production output for Ukraine that amounts to approximately 50% with a loss of more than half of its trading partners.

**Fig 5:**
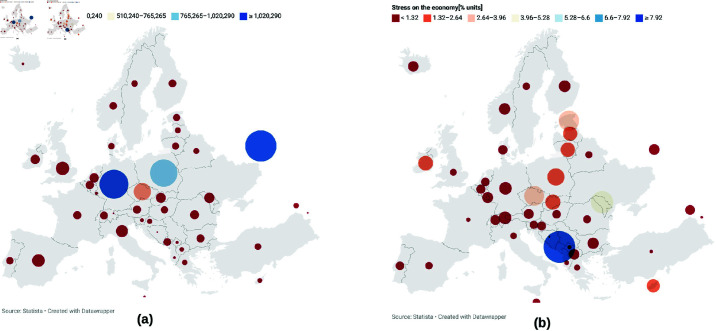
Statista data of refugees from Ukraine received by each country (a) (as of September 2023) and a map showing the stress exerted by these refugees on regional food sectors (b). The calculation was done by dividing the number of refugees by the total population of that region. The 2022 world population data of the World Bank was used. The maps were generated using the Datawrapper online tool found at https://www.datawrapper.de/.

**Fig 6:**
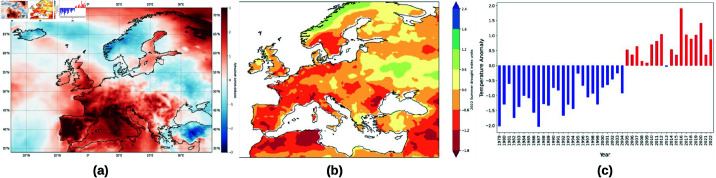
The 2022 July anomaly (a) compared to 1991–2022 climatology, (b) is the 2022 Summer drought index from the Global Crop Monitoring over Europe, and (c) is the yearly anomalies of Europe air temperature compared to the 1991 to 2022 average. These figures show the summer of 2022 was also one of the hottest on record. The plots were done using the ERA5 dataset from the Climate Data Store (CDS) of the Copernicus Climate Change Service (C3S) and data from the global drought monitoring system [72]. The maps were generated using Cartopy package in Python.

To quantify the contribution of the Ukrainian refugee crisis in Europe, we analyzed Statista’s refugee data for the year 2022–2023 (Fig 5). Fig 5(a) shows the number of refugees taken by each European country while Fig 5(b) shows the proportion of the number of refugees compared to the total population. The population data for 2022 from the World Bank was used. This figure shows that Germany, Poland, and the Czech Republic took in more refugees compared to France, Italy, and Spain. Considering the proportion of refugees to the total population, we find that countries near Ukraine such as Poland, Estonia, Lithuania, Moldova, and Slovakia were the most impacted by the influx of refugees. Similarly, we used the ERA5 surface temperature dataset from the Climate Data Store (CDS) of the Copernicus Climate Change Service (C3S) and data from the global drought monitoring system [72] to analyze and quantify the contribution of the extreme weather events of summer 2022 compared to climatology (Fig 6).

To shock the agricultural sectors of Ukraine, the EU, and Russia, we have developed damage function scenarios that take into account scenarios such as the invasion phase, the peak of the war, sanctions on Russia, Ukraine’s resistance, and the heatwaves and droughts of Summer 2022. Russia invaded Ukraine at the end of February 2022. Therefore, we consider March as the starting month for the losses in agricultural production and simulate the first year of hostilities, dividing the year into quarters, with the first quarter running from March to May (Invasion) and the second quarter from June to August (Peak). We assume that the peak of the war is in the 2nd quarter, while the scenario of international sanctions against Russia and Ukraine resistance takes place from the 3rd to the 4th quarter. The summer heatwave takes place in the 2nd quarter, and we sum up the impact of both events. Since Western Europe is more susceptible to weather extremes than Eastern Europe (as shown in Fig 6), we consider unevenly distributed shocks from extreme weather events. It is assumed that the impact of the war on the Russian food system is negligible in the first quarter, but peaks in the third and fourth quarters when Ukraine strikes back and sanctions take effect. The war-related shocks in other European countries are assumed to be caused by the continued influx of Ukrainian refugees and the stress that these refugees put on regional food banks.

**Fig 7:**
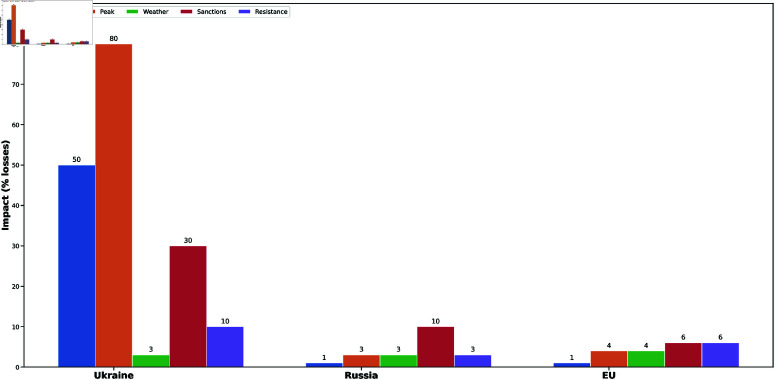
The distribution of shocks on the directly affected agricultural sectors due to the Russia-Ukraine war and the Summer weather extremes of 2022. The shock distributions give a higher weight on Ukraine’s agricultural sector compared to Russia, and the EU. The colored bars stand for the various scenarios under study. Blue for Invasion, Orange for Peak, Green for climate impacts, Red for Sanctions, and Purple for Ukraine’s resistance.

To distribute the shocks due to these two events, we assume a 50% decline in Ukraine’s agricultural production capacity in the inversion phase, an 80% decline in production capacity in the peak phase, 30% in the sanctions phase, and 10% in the resistance phase. For Russia, we assume a decline in agricultural production capacity of 1% in the inversion phase, 4% in the peak phase, 10% in the sanctions phase, and 3% in the resistance phase. For the EU, due to the influx of refugees and the strain on its agricultural system, we assume a decline in EU’s agricultural production capacity by 1% in the inversion phase, by 3% in the peak phase, by 6% in the sanctions phase and by 6% in the resistance phase, as the number of refugees continues to rise as the war continues, but levels off again over time. Fig 7 is a bar chart showing the distribution of shocks in the directly affected agricultural sectors due to the Russia-Ukraine war and the Summer weather extremes of 2022.

For the contribution of the extreme heatwaves of 2022, we consider the information published in the journal by Kornhuber *et al* [32]. They show that two or more weeks per summer spent under extreme heatwaves are associated with an average decline in crop production of 4% in the affected mid-latitude regions, with regional declines of up to 11%. We compared the surface temperature anomalies in the ERA5 dataset of the Copernicus Climate Change Service (C3S) Climate Data Store (CDS) with climatology and drought monitoring data from the Drought Monitoring System, which uses the ERA5 data, and contrasted the changes in anomalies and droughts between Eastern and Western Europe. Our analysis shows that Western Europe is more affected by extreme heatwaves and droughts compared to Eastern Europe and will therefore suffer high losses in agricultural production compared to Eastern Europe. Therefore, we assume a 4% decrease in agricultural production capacity due to extreme weather events in Western Europe compared to 3% in Eastern Europe. As the summer extreme events occur during the peak of the war, we add up their impact.

Using the damage function scenarios model as input to the demand-oriented, agent-based economic network model Acclimate, we shock the agricultural sectors of Ukraine, Russia, and the EU over one year in different seasons.

## 3 Results

**Fig 8:**
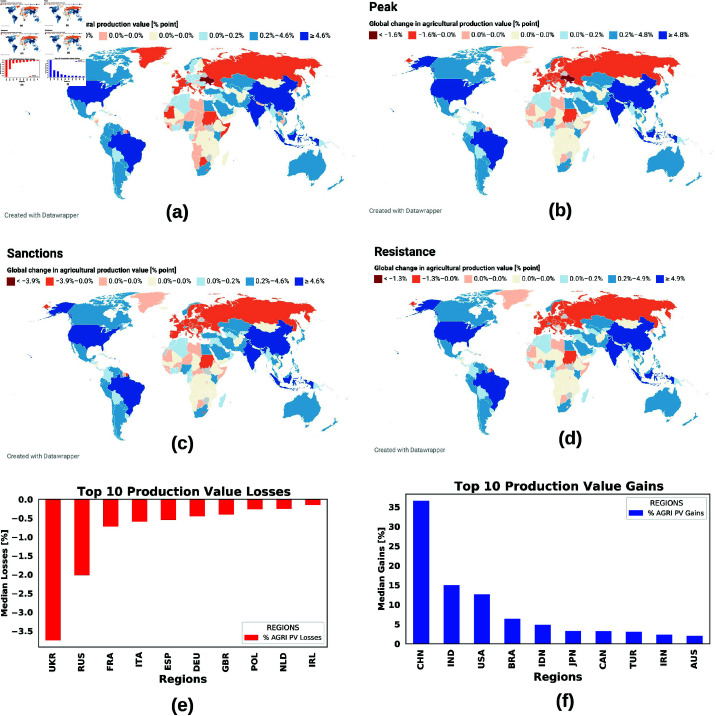
Global resonance of the Russia-Ukraine war on regional agricultural sectors. Scenarios such as the invasion, peak, sanctions, and Ukraine’s resistance to Russia are presented in (a, b, c, d) respectively. In (e) and (f), we present the top 10 most and least impacted countries due to the war. This figure shows that Southern European countries such as France, Italy, and Spain were hardest hit in Europe compared to the Northern neighbors, even though the direct impact of refugees on their food systems is relatively low. The USA and the majority of the BRICS countries, excluding Russia, are shown to be the least affected by the war while Sub-Saharan Africa appears to be the most impacted region with Russian allies such as Sudan, Central African Republic, and Niger, among the leading most affected regions. The maps were generated using the Datawrapper online tool found at https://www.datawrapper.de/.

The global resonance of agricultural production value losses due to the Russian-Ukrainian war is shown in Fig 8. Scenarios such as the invasion, the peak, sanctions on Russia, and Ukraine resistance are shown in a, b, c, and d respectively. In (e) and (f) we present the 10 countries most and least affected by the war when averaged over all scenarios under study. This figure shows that the agricultural production value of southern European countries such as France, Italy, and Spain were the most affected compared to their northern neighbors, although the direct impact of refugees on their food systems is relatively small. The USA and the majority of the BRICS countries (Brazil, India, China, and South Africa), except Russia, have been the least affected by the war. At the peak of the war, the global impact is greatest, followed by the Invasion phase. Tables 1 and 2 present the top 20 most and least impacted countries in terms of agricultural production value gains respectively. This table shows that sanctions on Russia with the war still escalating, will still lead to heavy production value losses in the EU. However, harsher sanctions on Russia coupled with Ukraine’s resistance will benefit the EU food markets, at the same time, the agricultural sectors of smaller nations and weaker economies become highly vulnerable.

**Table 1:** The top 20 most impacted countries in terms of agricultural production value losses for all scenarios under study. This table shows sanctions on Russia with the war still escalating, will still lead to heavy production value losses in the EU. However, with the application of harsher sanctions, the economies of smaller nations become highly vulnerable.

Rank	Invasion	Peak	Sanctions	Resistance	No refugees	Harsher sanctions
1	Ukraine	Ukraine	Russia	France	Ukraine	Russia
2	Russia	Russia	Ukraine	Russia	Russia	Ukraine
3	Italy	France	France	Italy	UAE	Liechtenstein
4	Spain	Spain	Italy	Spain	Pakistan	Sudan
5	UK	Italy	Spain	Germany	Holland	San Marino
6	Ireland	Germany	Germany	UK	Paraguay	Lesotho
7	France	UK	UK	Ukraine	Mozambique	Seychelles
8	Greece	Poland	Poland	Holland	Angola	Cabo Verde
9	Denmark	Holland	Holland	Poland	Cuba	N. Antilles
10	Latvia	Ireland	Ireland	Roumenia	Mauritius	Antigua
11	Malta	Greece	Denmark	Belgium	Botswana	Bermuda
12	Belgium	Belgium	Belgium	Denmark	Belize	Eritrea
13	Bulgaria	Denmark	Greence	Ireland	Bahamas	Sierra Leone
14	Liechtenstein	Roumania	Roumania	Greece	Somalia	Maldives
15	San Marino	Finland	Slovakia	Sweden	Jamaica	Greenland
16	Sudan	Portugal	Finland	Finland	Djibouti	Barbados
17	Lesotho	Czech	Czech	Czech	Barbados	Montenegro
18	Seychelles	Slovakia	Portugal	Slovakia	Maldives	Djibouti
19	Antigua	Hungary	Hungary	Hungary	Eritrea	New Caledonia
20	Bermuda	Sweden	Sweden	Austria	Montenegro	French Polynesia

**Table 2:** The top 20 least impacted countries in terms of agricultural production value gains for all scenarios under study. This table shows that the agricultural sectors of China, India, and the USA were the least impacted in 2022 during the weather-geopolitical couple extreme events. Harsher sanctions on Russia benefit the EU food markets but take a toll on weaker and smaller economies.

Rank	Invasion	Peak	Sanctions	Resistance	No refugees	Harsher sanctions
1	China	China	China	China	India	China
2	India	India	India	India	China	India
3	USA	USA	USA	USA	USA	USA
4	Brazil	Brazil	Brazil	Brazil	Germany	Brazil
5	Indonesia	Indonesia	Indonesia	Indonesia	France	Indonesia
6	Canada	Japan	Japan	Japan	Turkey	France
7	Turkey	Canada	Canada	Canada	Iran	Germany
8	Iran	Turkey	Turkey	Turkey	Italy	Italy
9	Japan	Iran	Iran	Iran	Holland	Japan
10	Australia	Australia	Mexico	Australia	Brazil	Canada
11	Mexico	Mexico	Australia	Mexico	Poland	Turkey
12	Kazakhstan	Korea	Kazakhstan	Thailand	Spain	Spain
13	Thailand	Thailand	Korea	Kazakhstan	Kazakhstan	Iran
14	Argentina	Kazakhstan	Thailand	Korea	Canada	Holland
15	Korea	Argentina	Argentina	Argentina	Hungary	Australia
16	Philippines	Philippines	Malaysia	Malaysia	Indonesia	Poland
17	Malaysia	Malaysia	Philippines	Philippines	Denmark	Mexico
18	S. Africa	S. Africa	S. Africa	S. Africa	Czech	Kazakhstan
19	Chile	Chile	Chile	New Zealand	Austria	UK
20	New Zealand	New Zealand	New Zealand	Chile	Slovakia	Thailand

**Fig 9:**
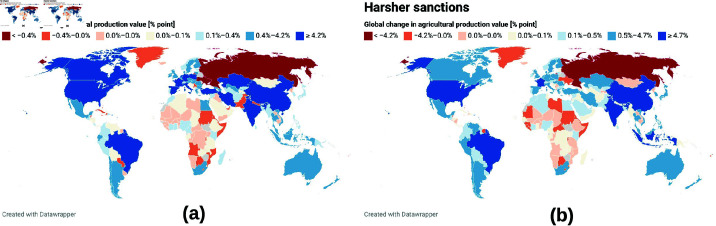
Global resonance of the Russia-Ukraine war on regional agricultural sectors. In this figure, we consider scenarios where the impact refugee crisis on European agricultural systems is ignored (a). In (b), we investigate the impact of harsher sanctions on Russia on global food sectors. This figure equally shows that harsh sanctions on Russia will hit Sub-Saharan Africa’s agricultural sectors harder than Europeans and others. The maps were generated using the Datawrapper online tool found at https://www.datawrapper.de/.

In Fig 9, we consider scenarios in which the impact of the refugee crisis on European agricultural systems is ignored (a), and in Fig 9(b) we analyze the impact of tougher sanctions against Russia on regional food sectors. Fig 9(a) and Tables 1 and 2 show that if we ignore the impact of refugees in the simulation phase, the EU agricultural sector shows positive growth during the war. This analysis is not entirely consistent with the reality on the ground. In the case where the impact of refugees is considered in the simulation phase (Table 1 column 1), the UK, Ireland, Southern European countries France, Italy, and Spain are shown to be the most affected, while the impact on smaller economies is lower compared to the scenario without refugee impact. Fig 9(b) shows the effects if tougher sanctions are imposed on Russia during the war and the refugee crisis is taken into account. This figure and Tables 1 and 2 show that tougher sanctions against Russia will hit the agricultural sector in sub-Saharan Africa and other weaker economies harder than the Europeans and others. Under these sanctions, European giants show a milder impact of the war while the Southeast European states such as Liechtenstein, Serbia, Bulgaria, Hungary, Croatia, etc. are the hardest hit countries in Europe.

**Fig 10:**
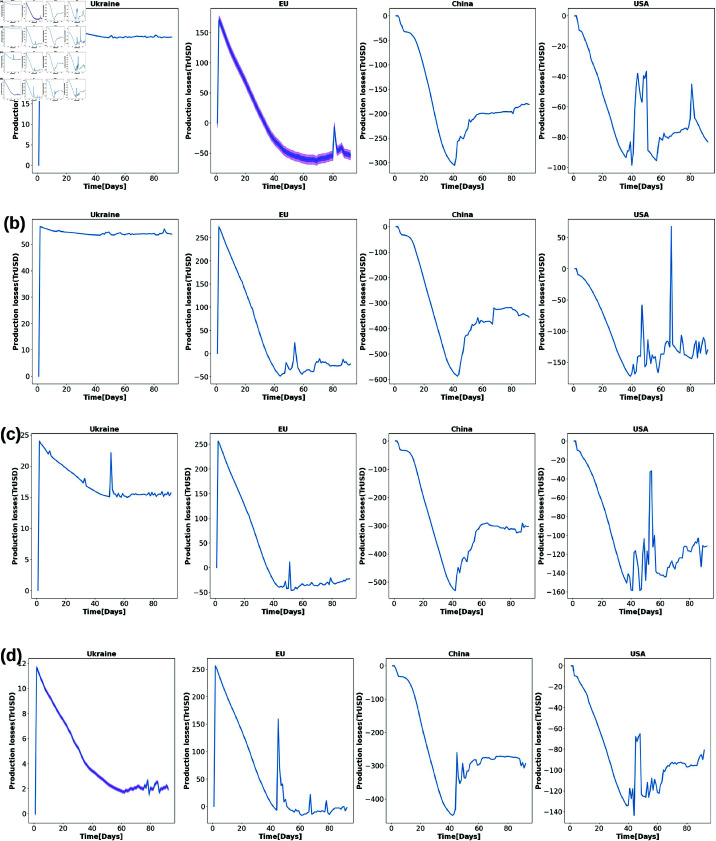
Shock induced by the Russia-Ukraine war on some key economies over the various scenarios studied. (a, b, c and d) are for the onset, peak, Ukrain fightback, and Sanctions on Russia respectively. Negative shocks imply a far much lesser impact. These figures show spiky shocks in the US agricultural sectors due to the war. We also observe a decrease in the impact of the war over time in the EU and Ukraine economies while China’s agricultural sectors observe some increase but remain relatively low.

Fig 10 shows the time series of shocks caused by the Russia-Ukraine war for some important economies in the various scenarios analyzed. (a, b, c and d) represent the invasion, the peak, the sanctions against Russia, and the resistance of Ukraine respectively. Negative shocks implied production value gains. These figures show spiky shocks in the US agricultural sectors due to the war with significant fluctuation in its agricultural production value. Overall, the production value stayed positive and high compared to that of the EU. For China, its agricultural production value experienced a boost over time followed by a significant decline but stayed positive. We also observe that the impact of the war in the EU and Ukraine decreases over time. While the EU agricultural sector is recovering over time, the Ukrainian sector continues to be negatively impacted, but with less impact. Analyzing the impact of the Russian-Ukrainian war on Ukraine’s global grain export data for 2020 (a) compared to 2022 (b) shows a drastic decline in the number of trading partners (Fig 3). The size of the bubble indicates the global share of production volume, the line thickness shows the volume of commodity flows and the color bar shows the continental locations of export partners. In this figure, we see that Ukraine can only export its product to Europe due to the war, while its production volume in 2022 has decreased significantly compared to 2020. Similarly, analyzing the impact of the Russian-Ukrainian war on Russia’s global grain exports for 2020 (a) compared to 2022 (b) shows that the sanctions against Russia have completely cut off its global grain network in 2022 compared to 2020 (Fig 4). This massive disruption could be the main cause of food shortages and price increases in sub-Saharan Africa and other weaker economies that are heavily dependent on Russia for food.

## 4 Discussion

With increasing globalization and international trade, the disruption of the supply chain in one region due to wars, weather extremes, and other disasters can have profound economic effects in another, distant region. How to mitigate and adapt to the impact of these shocks on regional economic sectors requires a thorough risk assessment of the economic impact of current and past events. A few studies have quantified the economic damage caused by the war between Russia and Ukraine. Ross *et al*. [73] analyzed the economic impact of the Russian-Ukrainian war on grain export disruption using the Global Trade Analysis Project (GTAP) computable general equilibrium model. Their results show a significant decline in Ukraine’s GDP compared to Russia’s, with the effects of the war spilling over to most regions of the world. This is a similar result to that shown in Fig 9 and in Tables 1 and 2. A report by the Asian Development Bank (ADB) shows strong GDP growth in the Caucasus and Central Asia shortly after the Russian invasion of Ukraine [23]. Another ADB [24] shows that this growth forecast has been lowered due to slower expansion in the People’s Republic of China (PRC). This is a similar result in the time series for China in Fig 10. Additionally, [74] investigated Countries’ vulnerability to food supply disruptions caused by the Russia–Ukraine war from a trade dependency perspective. By applying a set of trade and socioeconomic indicators, they found that the external food supplies of 279 countries and territories were affected to varying degrees; 24 countries—especially Georgia, Armenia, Kazakhstan, Azerbaijan, and Mongolia—are extremely vulnerable because they depend almost entirely on a variety of food imports from Russia and Ukraine. Moreover, they found that access to fertilizers was affected in 136 countries and territories, particularly Estonia (potassic fertilizer), Mongolia (nitrogenous fertilizers), Kazakhstan (mixed fertilizers), and Brazil, the United States, China, and India (all types of fertilizers). This study has equally shown that almost every country was affected by the Russia-Ukraine war while others were less impacted, those heavily relying on grain imports from Ukraine and Russia were the most impacted.

In Africa, the African Development Bank (AfDB) reported significant grain shortages due to the war in Ukraine. Muhammad *et al*. [75] reported in their paper that the sanctions imposed by Western countries on Russia have a spillover effect on the global economy, but did not show which countries are most affected by these sanctions. In this paper, we have shown the top 20 most and least affected agricultural sectors of the world under all scenarios. We equally showed that sub-Saharan countries and other weaker economies are most affected by the harsh sanctions against Russia, mainly due to their dependence on grain imports from the conflict regions. Most of the findings in the literature are consistent with the results of our simulation, which shows China, India, the US, and Brazil as some of the least affected countries during the war, and Sub-Saharan Africa and other weaker economies, especially those who allied with Russia as the most affected regions. Based on this risk assessment report, we strongly recommend that before drafting, adopting, and implementing economic sanctions against a conflict region (or several), the short- and long-term economic impact on its trading partners, especially those with weak economies, should be examined before these sanctions rebound on the regions imposing the sanctions. An example of this policy recommendation is the continuous renewal of the Black Sea Grain Agreement to reduce the impacts on economically vulnerable countries while stabilizing global food prices.

Some regional climate change mitigation strategies that should be implemented, particularly in Africa and other agricultural-producing countries, are to consider large-scale grain and food production to avoid over-reliance on imports. This approach involves massive support to local farmers by regional governments through incentives and the provision of agricultural tools and modern equipment geared towards increasing local food production. Local price control and the creation of new trading partners to reduce dependence on a single business partner can also help hedge against future disaster impacts. Another adaptation strategy is to shift consumer preferences towards cheaper and locally produced food.

### Limitations

Some drawbacks of this work are:

- The USDA data used might induce some inaccuracies during data collection, small farms might be underrepresented or ignored, and the survey sample may not represent the entire agricultural industry. Moreover, the WITS trade network data might not capture all trade routes. Using global climate model data to simulate climate-crops interaction, might not capture the actual interaction between the climate system and crop production.
- The war between Russia and Ukraine is still ongoing but not as intense as it was in 2022, so it is difficult to take stock of the economic damage caused by the war. That is why we considered a scenario-based modeling approach. Reliance on scenario-based modeling may not fully reflect the evolving situation and capture its complexity. (2) The USDA data used might induce some inaccuracies during data collection, small farms might be underrepresented or ignored, and the survey sample may not represent the entire agricultural industry. Moreover, the WITS trade network data might not capture all trade routes.

### Future work

In the future, we look to develop detailed shock models using factors affecting regional agricultural losses and real-time food market prices. We also look forward to investigating the ripple effects of the war on other economic sectors such as Oil and Gas, Transport, and Manufacturing. We will also try to model other highly probable geopolitical events such as the conflict between Russia and NATO, China and Taiwan, the USA and China, and Russia and the USA.

## 5 Conclusion

COVID-19 recovery plans have been thwarted for many countries, especially in Europe and Africa, following the full-scale invasion of Ukraine by Russia. At the same time, the war between Israel and Hamas, combined with Yemeni rebel attacks on ships in the Red Sea, and the disruption of a major global trade route, has put the entire Middle East at risk of a catastrophic humanitarian crisis since the Second World War. In addition, tensions between China and Taiwan are currently at an all-time high. These geopolitical events are taking place as the world grapples with the devastating effects of increasing weather extremes. These weather-geopolitical extreme events are currently exerting unprecedented shocks on the global food web in particular and the global economy in general, causing severe humanitarian crises such as the loss of lives, income inequality, food insecurity, and increasing poverty. Many studies have investigated the socio-economic impacts of extreme weather events especially on the regional agricultural sector. What has not been studied is the current threats posed by the co-existence of weather extremes and geopolitical conflicts, especially when a world major food producer is involved. This paper has presented the first risk assessment report on the economic impact of weather-geopolitical extreme events to raise awareness of the devastating economic crises posed by these events, contributing to early policy-making and sectoral intervention to mitigate or prevent future humanitarian crises.

Using the Russia-Ukraine war and the 2022 summer weather extremes as a case study, we developed a damage function scenario model for these events which serves as input for the demand-driven, agent-based economic model Acclimate. We have shown that the agricultural production values of Southern European countries such as France, Italy, and Spain were the most affected compared to their northern neighbors, although the direct impact of refugees on their food systems is relatively small. The US and most of the BRICS countries (Brazil, India, China, and South Africa), except Russia, are the least affected by the war, while sub-Saharan Africa and other weaker economies appear to be the most affected region, with Russian allies such as Sudan, the Central African Republic, and Niger among the most affected regions.

Furthermore, we have shown that in the case where the impact of refugees is considered during the simulation phase, the United Kingdom, Ireland, the Southern European states of France, Italy, and Spain are the most affected, while the impact on Sub-Saharan Africa is lower compared to the scenario without refugee impact. In the scenario in which tougher sanctions are imposed on Russia during the war with the refugee crisis is taken into account, the agricultural sectors in sub-Saharan Africa and other weaker economies are the hardest hit compared to those of the EU and other major economic powers. The south-eastern European states such as Serbia, Bulgaria, Hungary, Croatia, etc. are the most affected by these sanctions compared to the western European states.

A Black Sea Grain Agreement was signed in July 2022 as a policy to help reduce the global impact of the war by allowing Ukraine and Russia to export grains and fertilizers to the international markets. Researchers show that this agreement led to a significant drop in some food market prices. The agreement was extended three times before a fallout. Some trade agreements like the Black Sea Grain Initiative can help reduce the impact of wars on weaker economies and stabilize global food prices.

In conclusion, we strongly recommend prioritizing the economic impact on weaker and dependent economies when developing and adopting conflict measures to resolve global crises.

## References

[1] EP Briefing. EU sanctions on Russia: update, economic impact and outlook; 2023.

[2] Pisonero-Hernandez A, Muletier Z. Key findings of the 2023 Report on Ukraine; 2023.

[3] Arce O, Koester G, Nickel C. One year since Russia’s invasion of Ukraine – the effects on euro area inflation; 2023.

[4] Toh M, Ogura J, Humayun H, Yee I, Cheung E, Fossum S, et al. The list of global sanctions on Russia for the war in Ukraine; 2022.

[5] Funakoshi M, Lawson H, Deka K. Tracking sanctions against Russia; 2022.

[6] Wikipedia. International sanctions during the Russian invasion of Ukraine. 2023. Wikipedia. https://en.wikipedia.org/wiki/International_sanctions_during_the_Russian_invasion_of_Ukraine

[7] News B. West to cut some Russian banks off from Swift. 2022.

[8] News B. China State Banks Restrict Financing for Russian Commodities. 2022.

[9] Melander I, Baczynska G. EU targets Russian economy after ‘deluded autocrat’ Putin invades Ukraine; 2022.

[10] Liberty RFE. Western Countries Agree To Add Putin, Lavrov To Sanctions List; 2022.

[11] European Union. Infographic - EU solidarity with Ukraine; 2023.

[12] Shinozaki S. The Russian invasion of Ukraine and its impact on digitalized small firms in central and West Asia: evidence from rapid surveys. 2023.

[13] Katalin B, Tobias S. The surge in euro area food inflation and the impact of the Russia-Ukraine war. Econ Bullet. 2022;6(4).

[14] Jakob FA, Friderike K, Eliza ML, Tobias S. The impact of the war in Ukraine on euro area energy markets. Econ Bullet. 2022;1(4).

[15] Lorenz E, Michael F, Fausto P, Martin S. Euro area linkages with Russia: latest insights from the balance of payments. Economic Bulletin, 7, Box 8. 2022.

[16] Maria GA, Julia D, Rinalds G, Vanessa G, Michele M. Trade flows with Russia since the start of its invasion of Ukraine. Econ Bullet. 2022;1(5).

[17] Lane PR. Inflation Diagnostics; 2022.

[18] Evangelos C, Bruno F, Lukas H, Chiara O. The impact of the recent rise in inflation on low-income households. Econ Bullet. 2022;4(7).

[19] UNHCR. Ukraine refugee situation. 2023.

[20] Olga P, Olga T, Inna S, Olga B. How Ukrainian migrants affect the economies of European countries; 2023.

[21] Martins AM. Short‐term market impact of Black Sea Grain Initiative on four grain markets. J Futures Mark. 2024;44(4):619–30. doi: 10.1002/fut.22481

[22] Poursina D, Aleks Schaefer K, Hilburn S, Johnson T. Economic impacts of the Black Sea grain initiative. J Agricult Econ. 2023;75(1):457–64. doi: 10.1111/1477-9552.12549

[23] Hugot J. The economic impact of the Russian Invasion of Ukraine on the Caucasus and Central Asia: short-term benefits and long-term challenges; 2023.

[24] Outlook AD. Asian Development Outlook (ADO) 2022 Supplement: Recovery Faces Diverse Challenges; 2023.

[25] Yohannes-Kassahun B. One year later: the impact of the Russian conflict with Ukraine on Africa; 2023.

[26] Balima B. Huge protests in Niger call for French forces to leave after coup; 2023.

[27] Sarhadi A, Ausín MC, Wiper MP, Touma D, Diffenbaugh NS. Multidimensional risk in a nonstationary climate: joint probability of increasingly severe warm and dry conditions. Sci Adv. 2018;4(11):eaau3487. doi: 10.1126/sciadv.aau3487 30498780 PMC6261656

[28] Lehmann J, Coumou D, Frieler K. Increased record-breaking precipitation events under global warming. Climatic Change. 2015;132(4):501–15. doi: 10.1007/s10584-015-1434-y

[29] Schär C, Vidale PL, Lüthi D, Frei C, Häberli C, Liniger MA, et al. The role of increasing temperature variability in European summer heatwaves. Nature. 2004;427(6972):332–6. doi: 10.1038/nature02300 14716318

[30] Zscheischler J, Seneviratne SI. Dependence of drivers affects risks associated with compound events. Sci Adv. 2017;3(6):e1700263. doi: 10.1126/sciadv.1700263 28782010 PMC5489265

[31] Kornhuber K, et al. Extreme weather events in early summer 2018 connected by a recurrent hemispheric wave-7 pattern. Environ Res Lett. 2019;14:054002.

[32] Kornhuber K, Coumou D, Vogel E, Lesk C, Donges JF, Lehmann J, et al. Amplified Rossby waves enhance risk of concurrent heatwaves in major breadbasket regions. Nat Clim Chang. 2019;10(1):48–53. doi: 10.1038/s41558-019-0637-z

[33] Kornhuber K, Petoukhov V, Petri S, Rahmstorf S, Coumou D. Evidence for wave resonance as a key mechanism for generating high-amplitude quasi-stationary waves in boreal summer. Clim Dyn. 2017;491961–79.

[34] Screen JA, Simmonds I. Amplified mid-latitude planetary waves favour particular regional weather extremes. Nat Clim Change. 2014;4(8):704–9. doi: 10.1038/nclimate2271

[35] Coumou D, Petoukhov V, Rahmstorf S, Petri S, Schellnhuber HJ. Quasi-resonant circulation regimes and hemispheric synchronization of extreme weather in boreal summer. Proc Natl Acad Sci U S A. 2014;111(34):12331–6. doi: 10.1073/pnas.1412797111 25114245 PMC4151761

[36] Deng K, Yang S, Ting M, Lin A, Wang Z. An intensified mode of variability modulating the summer heat waves in Eastern Europe and Northern China. Geophys Res Lett. 2018;45361–9.

[37] Lau WKM, Kim KM. The 2010 Pakistan Flood and Russian heat wave: teleconnection of hydrometeorological extremes. J Hydrometeorol. 2012;13392–403.

[38] Saeed S, Van Lipzig N, Müller WA, Saeed F, Zanchettin D. Influence of the circumglobal wave-train on European summer precipitation. Clim Dyn. 2014; 43503–15.

[39] Petoukhov V, Rahmstorf S, Petri S, Schellnhuber HJ. Quasiresonant amplification of planetary waves and recent Northern Hemisphere weather extremes. Proc Natl Acad Sci U S A. 2013;110(14):5336–41. doi: 10.1073/pnas.1222000110 23457264 PMC3619331

[40] Mann ME, Rahmstorf S, Kornhuber K, Steinman BA, Miller SK, Coumou D. Influence of anthropogenic climate change on planetary wave resonance and extreme weather events. Sci Rep. 2017;7:45242. doi: 10.1038/srep45242 28345645 PMC5366916

[41] Mora C, Spirandelli D, Franklin EC, Lynham J, Kantar MB, Miles W, et al. Broad threat to humanity from cumulative climate hazards intensified by greenhouse gas emissions. Nat Clim Change. 2018;8(12):1062–71. doi: 10.1038/s41558-018-0315-6

[42] Lobell DB, Schlenker W, Costa-Roberts J. Climate trends and global crop production since 1980. Science. 2011;333(6042):616–20. doi: 10.1126/science.1204531 21551030

[43] Lobell DB, Burke MB. Why are agricultural impacts of climate change so uncertain? The importance of temperature relative to precipitation. Environ Res Lett. 2008;3(3):034007. doi: 10.1088/1748-9326/3/3/034007

[44] Arreyndip NA. Identifying agricultural disaster risk zones for future climate actions. PLoS One. 2021;16(12):e0260430. doi: 10.1371/journal.pone.0260430 34855827 PMC8638849

[45] Vogel E, Donat MG, Alexander LV, Meinshausen M, Ray DK, Karoly D, et al. The effects of climate extremes on global agricultural yields. Environ Res Lett. 2019;14(5):054010. doi: 10.1088/1748-9326/ab154b

[46] Connors JPC, Janetos A, Romitti Y. Agricultural losses in a telecoupled world. Extreme Events Climate Change. 2021;67–88. doi: 10.1002/9781119413738.ch5

[47] Stevanović M, Popp A, Lotze-Campen H, Dietrich JP, Müller C, Bonsch M, et al. The impact of high-end climate change on agricultural welfare. Sci Adv. 2016;2(8):e1501452. doi: 10.1126/sciadv.1501452 27574700 PMC4996644

[48] Burkholz R, Schweitzer F. International crop trade networks: the impact of shocks and cascades. Environ Res Lett. 2019;14(11):114013. doi: 10.1088/1748-9326/ab4864

[49] Schaub S, Finger R. Effects of drought on hay and feed grain prices. Environ Res Lett. 2020;15(3):034014. doi: 10.1088/1748-9326/ab68ab

[50] Bren d’Amour C, Wenz L, Kalkuhl M, Christoph Steckel J, Creutzig F. Teleconnected food supply shocks. Environ Res Lett. 2016;11(3):035007. doi: 10.1088/1748-9326/11/3/035007

[51] Porfirio LL, Newth D, Finnigan JJ, Cai Y. Economic shifts in agricultural production and trade due to climate change. Palgrave Commun. 2018;4(1). doi: 10.1057/s41599-018-0164-y

[52] Moore FC, et al. Economic impacts of climate change on agriculture: a comparison of process-based and statistical yield models Environ Res Lett. 2017; 12:065008.

[53] Corbeels M, Berre D, Rusinamhodzi L, Lopez-Ridaura S. Can we use crop modelling for identifying climate change adaptation options? Agricult Forest Meteorol. 2018;256–257:46–52. doi: 10.1016/j.agrformet.2018.02.026

[54] Davis KF, Downs S, Gephart JA. Towards food supply chain resilience to environmental shocks. Nat Food. 2021;2(1):54–65. doi: 10.1038/s43016-020-00196-3 37117650

[55] De Winne J, Peersman G. The adverse consequences of global harvest and weather disruptions on economic activity. Nat Clim Chang. 2021;11(8):665–72. doi: 10.1038/s41558-021-01102-w

[56] Duchenne-Moutien RA, Neetoo H. Climate change and emerging food safety issues: a review. J Food Prot. 2021;84(11):1884–97. doi: 10.4315/JFP-21-141 34185849

[57] Fan S, Cho EE, Meng T, Rue C. How to prevent and cope with coincidence of risks to the global food system. Annu Rev Environ Resour. 2021;46(1):601–23. doi: 10.1146/annurev-environ-012220-020844

[58] Folberth C, Skalský R, Moltchanova E, Balkovič J, Azevedo LB, Obersteiner M, et al. Uncertainty in soil data can outweigh climate impact signals in global crop yield simulations. Nat Commun. 2016;7:11872. doi: 10.1038/ncomms11872 27323866 PMC4919520

[59] Jägermeyr J, Müller C, Ruane AC, Elliott J, Balkovic J, Castillo O, et al. Climate impacts on global agriculture emerge earlier in new generation of climate and crop models. Nat Food. 2021;2(11):873–85. doi: 10.1038/s43016-021-00400-y 37117503

[60] Malesios C, Jones N, Jones A. A change-point analysis of food price shocks. Climate Risk Manag. 2020;27:100208. doi: 10.1016/j.crm.2019.100208

[61] Nelson GC, van der Mensbrugghe D, Ahammad H, Blanc E, Calvin K, Hasegawa T, et al. Agriculture and climate change in global scenarios: why don’t the models agree. Agricult Econ. 2013;45(1):85–101. doi: 10.1111/agec.12091

[62] Rötter RP, Appiah M, Fichtler E, Kersebaum KC, Trnka M, Hoffmann MP. Linking modelling and experimentation to better capture crop impacts of agroclimatic extremes—a review. Field Crops Res. 2018;221:142–56. doi: 10.1016/j.fcr.2018.02.023

[63] Magalhães Vital T, Dall’erba S, Ridley W, Wang X. What do the 235 estimates from the literature tell us about the impact of weather on agricultural and food trade flows?. Glob Food Secur. 2022;35:100654. doi: 10.1016/j.gfs.2022.100654

[64] Waldhoff ST, Wing IS, Edmonds J, Leng G, Zhang X. Future climate impacts on global agricultural yields over the 21st century. Environ Res Lett. 2020;15(11):114010. doi: 10.1088/1748-9326/abadcb

[65] Xie W, Cui Q, Ali T. Role of market agents in mitigating the climate change effects on food economy. Nat Hazards. 2019;99(3):1215–31. doi: 10.1007/s11069-019-03646-9

[66] Willner SN, Otto C, Levermann A. Global economic response to river floods. Nat Clim Change. 2018;8(7):594–8. doi: 10.1038/s41558-018-0173-2

[67] Kuhla K, Willner SN, Otto C, Wenz L, Levermann A. Future heat stress to reduce people’s purchasing power. PLoS One. 2021;16(6):e0251210. doi: 10.1371/journal.pone.0251210 34111129 PMC8191966

[68] Kuhla K, Willner SN, Otto C, Geiger T, Levermann A. Ripple resonance amplifies economic welfare loss from weather extremes. Environ Res Lett. 2021;16(11):114010. doi: 10.1088/1748-9326/ac2932

[69] Puma MJ, et al. Assessing the evolving fragility of the global food system. Environ Res Lett. 2015;10:024007.

[70] Otto C, Willner SN, Wenz L, Frieler K, Levermann A. Modeling loss-propagation in the global supply network: the dynamic agent-based model acclimate. J Econ Dyn Control. 2017;83:232–69. doi: 10.1016/j.jedc.2017.08.001

[71] Wenz L, Levermann A. Enhanced economic connectivity to foster heat stress-related losses. Sci Adv. 2016;2(6):e1501026. doi: 10.1126/sciadv.1501026 27386555 PMC4928955

[72] Vicente‐Serrano SM, Domínguez‐Castro F, Reig F, Tomas‐Burguera M, Peña‐Angulo D, Latorre B, et al. A global drought monitoring system and dataset based on ERA5 reanalysis: a focus on crop‐growing regions. Geosci Data J. 2022;10(4):505–18. doi: 10.1002/gdj3.178

[73] Rose A, Chen Z, Wei D. The economic impacts of Russia–Ukraine War export disruptions of grain commodities. Appl Eco Perspect Pol. 2023;45(2):645–65. doi: 10.1002/aepp.13351 PMC12045327 40313862

[74] Zhang Z, Abdullah MJ, Xu G, Matsubae K, Zeng X. Countries’ vulnerability to food supply disruptions caused by the Russia–Ukraine war from a trade dependency perspective. Sci Rep. 2023;13(1):16591. doi: 10.1038/s41598-023-43883-4 PMC10547748 37789089

[75] Balbaa ME, Eshov MP, Ismailova N. The Impacts of Russian Ukrainian War on the Global Economy in the frame of digital banking networks and cyber attacks. In: Proceedings of the 6th International Conference on Future Networks and Distributed Systems. 2022. p. 137–46. doi: 10.1145/3584202.3584223

